# Human Plasma and Recombinant Hemopexins: Heme Binding Revisited

**DOI:** 10.3390/ijms22031199

**Published:** 2021-01-26

**Authors:** Elena Karnaukhova, Catherine Owczarek, Peter Schmidt, Dominik J. Schaer, Paul W. Buehler

**Affiliations:** 1 Center for Biologics Evaluation and Research, Food and Drug Administration, Silver Spring, MD 20993, USA; 2 CSL Limited, Bio21 Institute, Parkville, Victoria 3010, Australia; catherine.owczarek@csl.com.au (C.O.); Peter.Schmidt@csl.com.au (P.S.); 3 Division of Internal Medicine, University Hospital of Zurich, 8091 Zurich, Switzerland; dominik.schaer@usz.ch; 4 Department of Pathology, The University of Maryland School of Medicine, Baltimore, MD 21201, USA; 5 The Center for Blood Oxygen Transport and Hemostasis, Department of Pediatrics, The University of Maryland School of Medicine, Baltimore, MD 21201, USA

**Keywords:** heme, hemopexin, circular dichroism, recombinant hemopexin

## Abstract

Plasma hemopexin (HPX) is the key antioxidant protein of the endogenous clearance pathway that limits the deleterious effects of heme released from hemoglobin and myoglobin (the term “heme” is used in this article to denote both the ferrous and ferric forms). During intra-vascular hemolysis, heme partitioning to protein and lipid increases as the plasma concentration of HPX declines. Therefore, the development of HPX as a replacement therapy during high heme stress could be a relevant intervention for hemolytic disorders. A logical approach to enhance HPX yield involves recombinant production strategies from human cell lines. The present study focuses on a biophysical assessment of heme binding to recombinant human HPX (rhHPX) produced in the Expi293F^TM^ (HEK293) cell system. In this report, we examine rhHPX in comparison with plasma HPX using a systematic analysis of protein structural and functional characteristics related to heme binding. Analysis of rhHPX by UV/Vis absorption spectroscopy, circular dichroism (CD), size-exclusion chromatography (SEC)-HPLC, and catalase-like activity demonstrated a similarity to HPX fractionated from plasma. In particular, the titration of HPX apo-protein(s) with heme was performed for the first time using a wide range of heme concentrations to model HPX–heme interactions to approximate physiological conditions (from extremely low to more than two-fold heme molar excess over the protein). The CD titration data showed an induced bisignate CD Soret band pattern typical for plasma and rhHPX versions at low heme-to-protein molar ratios and demonstrated that further titration is dependent on the amount of protein-bound heme to the extent that the arising opposite CD couplet results in a complete inversion of the observed CD pattern. The data generated in this study suggest more than one binding site in both plasma and rhHPX. Furthermore, our study provides a useful analytical platform for the detailed characterization of HPX–heme interactions and potentially novel HPX fusion constructs.

## 1. Introduction

Heme (iron-protoporphyrin IX) is transferred from extra-cellular hemoglobin (Hb) into plasma proteins and lipids or tissues under numerous clinical conditions and medical interventions [1,2,3,4,5]. Hemopexin (HPX) is an acute-phase plasma glycoprotein (~57 kD) whose major physiological function is the binding and clearance of heme from the central compartment. HPX binds heme with the highest affinity of any known protein (K_d_ < 1 pM) to form a low-spin bis-histidyl HPX/heme equimolar complex, thus preventing heme transfer and toxicological response, while facilitating cellular clearance and metabolism [6,7,8,9,10]. HPX operates as a second line of endogenous defense against heme-mediated oxidative stress following the depletion of haptoglobin by extra-cellular Hb [2,3,11,12].

The physiological plasma levels of HPX are reported to be 1–2 mg/mL [1]. However, under pathological hemolytic conditions, when the concentration of bioavailable heme in the plasma exceeds circulatory binding capacity, HPX levels can become acutely reduced [1,13,14,15]. Reduced plasma concentrations of HPX are associated with high plasma levels of albumin-, apo-lipoprotein- and tissue-heme oxidation products in patients with hemolytic disorders [16].

Therefore, HPX replacement therapy has been proposed as a promising approach to control pathophysiology following high heme stress [4,17,18,19,20,21,22]. Currently, there are no approved HPX-based therapeutic preparations available in the US, nor abroad. Early development efforts have focused on plasma HPX; nonetheless, recombinant HPX versions may offer cost-effective approaches to developing HPX and fusion-based HPX therapeutics. The characterization and evaluation of the heme-scavenging potential of novel HPX preparations compared to plasma HPX is essential to early development, prior to costly animal and clinical studies. Several systems have been used to express human gene for HPX; however, recombinant forms of HPX remain essentially uncharacterized, leaving scientific gaps in our understanding of protein folding, heme binding, and structural integrity after expression to allow for comparisons with plasma-derived HPX [12]. The primary focus of the study presented herein is a biophysical assessment of heme binding to recombinant human HPX (rhHPX) produced in an Expi293F^TM^ (HEK293) mammalian cell system compared to plasma HPX. 

Our UV/Vis, circular dichroism (CD), size-exclusion chromatography (SEC), catalase-like activity assay, and temperature-induced unfolding data demonstrated a close similarity between rhHPX and its human plasma counterpart. However, a secondary focus arose from results suggesting that our titration of rhHPX with heme revealed dramatic changes in the protein-induced chirality profile. This experimental observation is strongly dependent on the amount of protein-bound heme and prompted us to revisit heme interactions with both plasma HPX and rhHPX in comparative experiments.

The heme-binding capacity of HPX was evaluated as one mole of heme per one mole of HPX [23], which was further supported by a crystal structure of the HPX/heme complex that revealed a high-affinity heme-binding site formed between two beta-propeller domains of the protein [24]. Not surprisingly, only the preparation of stoichiometric 1:1 HPX/Heme complexes are reported [24,25,26,27,28,29,30,31,32]; however, the equimolar complexes reveal a striking variety of induced CD spectral data [32,33,34,35].

Within this component of the study, we performed a titration of rhHPX (as well as plasma HPX) with heme across a wide range of ligand-to-protein molar ratios from 0.1 up to 2.4. This approach differs from other CD studies that mainly characterized equimolar complexes of heme (or mesoheme) with human, rabbit, or murine HPX that were prepared by the incubation of protein and ligand (at an equimolar or higher heme content) followed by dialysis to remove the excess ligand. The purpose here was to evaluate the pattern of protein-induced CD of heme in rhHPX/Heme complexes that can be used as a reference for the characterization of heme-binding studies. The data generated by the titration of rhHPX and plasma HPX with heme, together with catalase-like activity, thermal unfolding, and SEC-HPLC data, suggest that HPX interactions with heme are not limited to a single heme-binding site, but rather suggest a secondary site of low affinity, which can bind an additional amount of heme. The methodology developed in this study, and specifically rhHPX/heme titration data with a characteristic induced CD pattern that strongly depends on the heme level in the sample, may be applicable to evaluating the performance of novel HPX constructs, as well as for a better interpretation of currently available data.

## 2. Results and Discussion

### 2.1. Characterization of rhHPX

**Figure 1:**
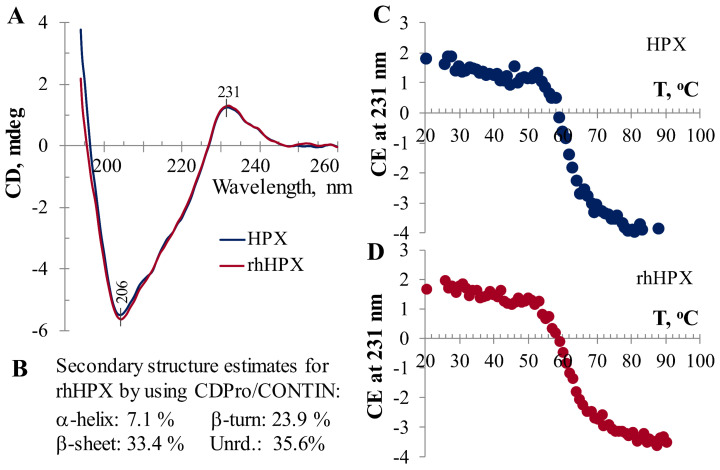
Far-UV circular dichroism (CD) data for plasma hemopexin (HPX) and recombinant human HPX (rhHPX). Overlay of far-UV CD spectra (**A**), rhHPX secondary structure estimates (**B**), and temperature-induced unfolding (**C**,**D**) suggest a close folding similarity between rhHPX and its plasma fractionated counterpart.

Prior to conducting heme-binding experiments, we evaluated rhHPX in comparison with plasma HPX, which is used as a reference, for folding by far-UV CD (Figure 1) and molecular integrity by SEC-HPLC (*vide infra*). As shown in Figure 1, rhHPX exhibits a characteristic far-UV CD spectrum with a close similarity to that of plasma HPX showing a strong negative extremum at 206 nm and a characteristic positive CD band at 231–232 nm. The ellipticity at 231–232 nm is attributed to through-space interactions between the chromophoric tryptophan residues and considered to be a sensitive marker of the protein correct folding [36,37,38,39,40,41,42].

**Figure 2:**
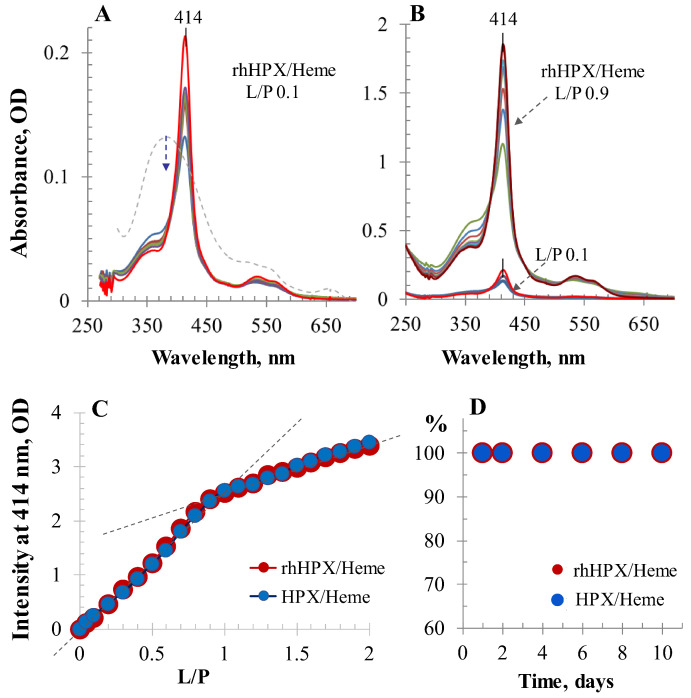
UV/Vis data for heme binding to rhHPX and plasma HPX. (**A**) Dynamics of heme binding to rhHPX at ligand-to-protein ratio (L/P) 0.1 monitored during 10 min (gray dotted trace shows heme in phosphate buffer saline (PBS) alone). (**B**) Time-course UV/Vis spectra of rhHPX/Heme at L/P 0.9 collected during 10 min (overlaid with A). (**C**) The absorbance intensities at 414 nm of heme complexes with rhHPX and plasma HPX plotted vs. L/P. Protein concentration for both rhHPX and plasma HPX was 18 μM in PBS. All UV/Vis data were collected in differential form by measuring spectra of mixed samples against respective 18 μM protein in a 10 mm path length cuvette. The samples with the absorbance intensity at 414 nm exceeding the instrument absorbance accuracy limit (higher than 2.4 OD) were measured in a 5 mm pathlength cuvette. (**D**) Monitoring stability of the heme complexes with HPX and rhHPX by the Soret band intensity (shown for samples with L/P 1).

The far-UV CD spectra of plasma HPX and rhHPX (Figure 1A) were used to estimate the protein secondary structure [43]. According to the percentage of secondary structure elements we obtained by using CDPro/CONTIN (Figure 2B), the secondary structure of both HPX and rhHPX is dominated by β-structures (57.3–57.6%) and demonstrates a low content of α-helices (~7%), which is consistent with crystal structure data [24].

The similarity of rhHPX folding to that of plasma HPX was further supported by thermal unfolding data (Figure 1C,D). Although the temperature-induced unfolding (melting) profiles recorded for rhHPX and plasma HPX are not identical, the melting points are approximately 62 ± 0.5 °C, within accuracy of the experiments.

### 2.2. UV/Vis Titration

Interactions of plasma HPX and rhHPX with heme were investigated using UV/Vis absorption spectroscopy and then by visible CD. Figure 2A shows UV/Vis differential spectra for an 18 μM solution of rhHPX in phosphate buffer saline (PBS) recorded immediately after adding a 0.1-equivalent aliquot of heme. While the heme added to PBS alone is characterized by a broad spectrum with a maximum absorption around 370 nm of decreasing intensity (broken gray trace), the mixed sample rhHPX/Heme with ligand-to-protein (molar) ratio (L/P) 0.1 immediately exhibits a sharp Soret band at 413.5–414 nm with an intensity that rapidly increases within a 10-min timeframe and minimal changes thereafter. The titration of plasma HPX and rhHPX with heme was conducted in parallel. Each step of the UV/Vis titration was monitored for at least 40 min to ensure that the ligand–protein interaction reached completion. Figure 2B reflects similar dynamics for rhHPX/Heme complexes with higher heme content (shown here for L/P 0.9) and demonstrates a characteristic isosbestic point typical for UV/Vis spectral changes of each intermediate.

UV/Vis titration data for HPX/Heme and rhHPX/Heme are summarized in Figure 2C as the Soret band intensity versus heme content in the samples (up to L/P 2) showing two different UV/Vis intensity slopes that demonstrate similar heme complex formation with each protein. Similar UV/Vis titration profiles were earlier reported for rat, rabbit, and human plasma-derived HPX preparations [23,37,44] indicating HPX heme-binding capacity to be greater than an equimolar amount of heme. 

Figure 2D illustrates the exceptional stability of rhHPX/Heme and HPX/Heme that was evaluated by monitoring the Soret band intensities of protein/heme equimolar samples during 10-day storage at room temperature.

### 2.3. Heme Transfer from Methemalbumin to HPX

**Figure 3:**
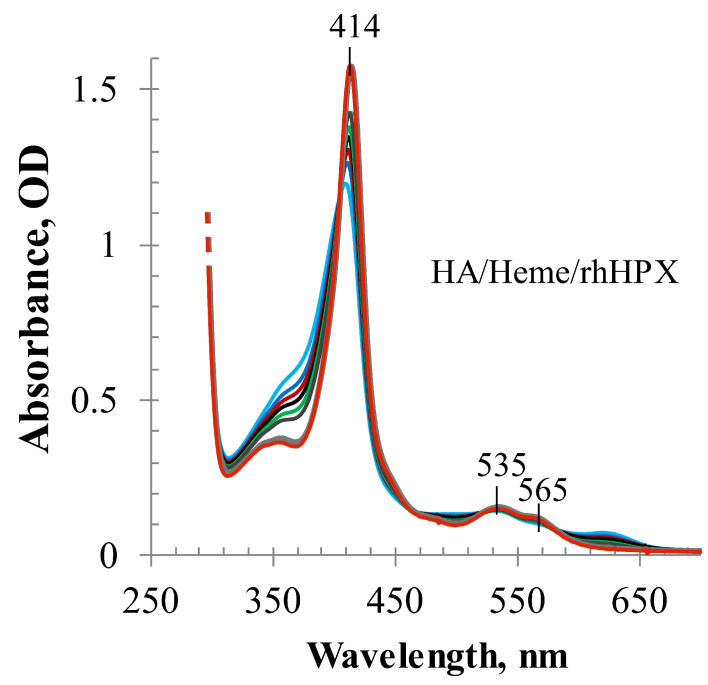
UV-Vis spectra of heme transfer from methemalbumin (HA/Heme) to rhHPX monitored for 10 min after adding rhHPX (light blue trace corresponds to 1 min, and red trace—to 10 min).

The heme-binding properties of rhHPX were further studied in experiments to assess heme transfer from the human albumin heme complex (methemalbumin) (HA/Heme at L/P 1, λ_max_ at 403 nm) to rhHPX. As reflected in Figure 3, heme transfer from methemalbumin to rhHPX began immediately after the addition of rhHPX to HA/Heme sample and was observed as a sharp increase in the Soret band at a λ_max_ at 413.5–414 nm.

However, it takes approximately 24 h to reach the state of no further changes in the absorption spectra of the mixed sample, similar to that reported for plasma HPX [6]. The bathochromic shift of 11-nm in the λ_max_ position, from 403 nm of HA/Heme to 414 nm of HA/HPX/Heme mixed sample, indicates that rhHPX efficiently transfers heme from methemalbumin to generate a stable rhHPX/Heme complex.

### 2.4. CD Titration

Heme interactions with plasma HPX and rhHPX were further investigated by CD spectroscopy in the wavelength range that would allow for monitoring of heme–protein complex formation, i.e., by monitoring the induced Soret band CD changes (350–500 nm). Porphyrins are powerful and highly sensitive chromophores that are exceptionally amenable to CD spectroscopy analysis because of the intensities of chromophoric absorption facilitated by their high extinction coefficients [45,46,47,48]. The Soret band region of the CD spectrum provides the most informative insight into the heme interactions with HPX at the protein binding site(s). Heme is an achiral molecule, and as an optically inactive compound, it does not exhibit any significant CD signal of its own. However, being situated in the protein binding site, heme behaves as an optically active compound (a phenomenon of protein-induced chirality). Protein-bound heme may exhibit a prominent Cotton Effect (CE) in the region of the Soret band and some optical activity in the visible range. Optical activity in the Soret region of hemoproteins occurs based on the coupling of the heme ππ* electronic dipole transition moments with those of proximate aromatic amino acid residues (mainly, Trp) of the protein matrix and heme distortions from planarity [49,50,51]. The origin of the optical activity in the Soret region has been extensively investigated [52,53,54,55]. Therefore, characteristic induced CD (ICD) spectra of hemoproteins (e.g., hemoglobin, myoglobin) or other heme-binding proteins are indicative of nonplanar distortions of the heme and its association with protein. 

Heme equimolar complexes with HPX (rabbit, rat, and human) are well assessed by Soret CD [26,27,28,29,30,31,32], yet there are discrepancies in the CD data across these studies. Along with monophasic (monolobe) ICD [33], various biphasic (also called bisignate or bilobe) ICD spectra were published for heme complexes with plasma HPX [32,34,35].

The intriguing fact that a single heme-sited HPX [24] exhibits a strong bisignate CD related to the Soret band has been a point of discussion and debate. According to one plausible interpretation, human plasma HPX preparations are comprised of two spectroscopically distinct, non-interconverting HPX forms that exhibit characteristically different Soret CD spectra, which superimposition is observed as a bisignate spectrum [35]. In general, bisignate ICD spectra observed for a single heme-liganded HPX can be explained via an exciton coupling between two or more chromophores that may occur in the case of HPX dimerization and/or aggregation or using heme stock solutions containing heme dimers/aggregates. 

**Figure 4:**
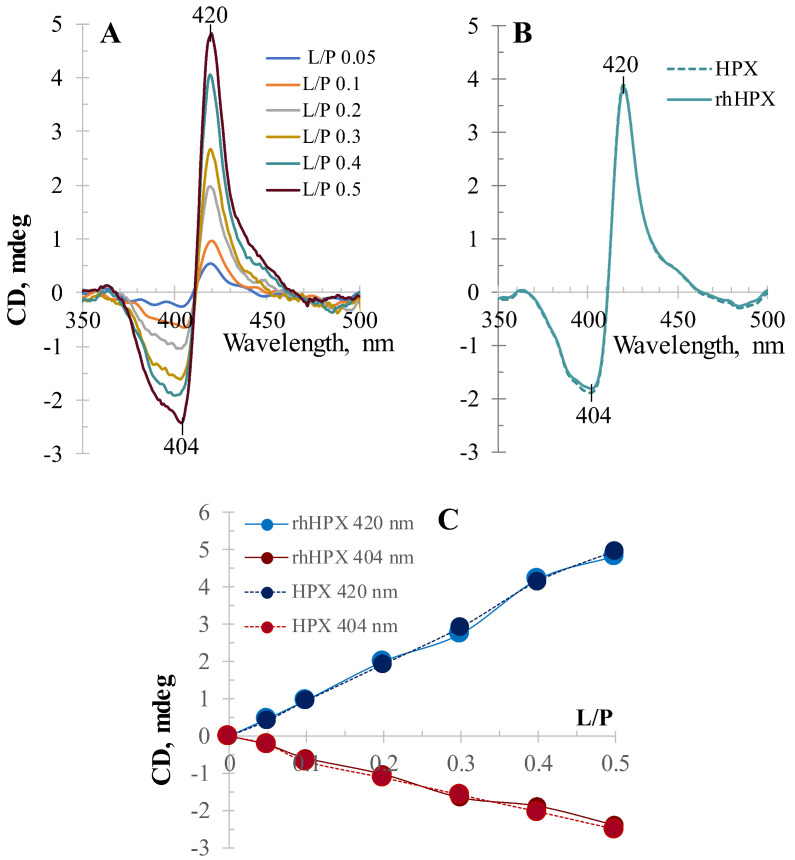
The CD titration of rhHPX and plasma HPX with heme in a low L/P range (from 0.05 to 0.5) shows closely comparable patterns: (**A**) Plasma HPX; (**B**) Induced CD for heme complexes with plasma HPX and rhHPX of the same L/P are similar (shown for L/P 0.4). (**C**) The intensities of the 1st CE (420 nm) and 2nd CE (404 nm) plotted versus L/P values up to 0.5 for rhHPX/Heme (dark blue and purple circles, respectively) and plasma HPX/Heme (respective lighter shades).

Figure 4A shows the CD titration of rhHPX with heme in a low L/P range, which was performed with starting heme content in the sample as low as 0.05 molar equivalent relative to the rhHPX concentration to specifically evaluate whether it may result in a monolobe single-CE CD spectrum, which is expected from a protein with a single heme-binding site. However, even heme binding at this low ligand-to-protein ratio did not produce a clear monolobe CD, showing the first positive CE at 420 nm and indication of a weak second CE around 404 nm (blue trace). At a 1:10 (L/P 0.1) heme-to-protein stoichiometry, the bisignate ICD pattern is clearly observed. Further rhHPX titration with heme (shown here up to an L/P of 0.5) resulted in a proportional increase of the ICD intensities at 420 nm (1st positive CE) and at 404 nm (2d negative CE) that could be explained by exciton coupling between heme bound to rhHPX at two sites. It is noteworthy that all six CD spectra shown on plot A intercept at an identical isosbestic point (411–412 nm) in good correlation with the Soret band absorption λ_max_, which is in accordance with the exciton coupling theory [45,46,48]. However, the bisignate ICD pattern could also be interpreted because of heme binding to two distinct forms of protein as proposed for human plasma HPX [35]. We certainly expected that exploring a recombinant platform must result in a uniform recombinant protein and exclude a possibility of protein isoforms with differing conformational arrangement for heme binding. Nevertheless, these titrations of rhHPX and plasma HPX were performed in parallel and demonstrate similar bisignate CD titration patterns. Figure 4B shows an overlay of ICD spectra for rhHPX/Heme and plasma HPX/Heme (both at an L/P 0.4) to illustrate a close similarity observed for the titration intermediate samples. Figure 4C provides a comparative summary for rhHPX and plasma HPX titration with heme at relatively low heme-to-protein ratios, demonstrating the similarity between the two proteins and an almost linear increase in the intensities of positive and negative ICD bands at 420 nm and 404 nm versus the content of heme in the samples.

**Figure 5:**
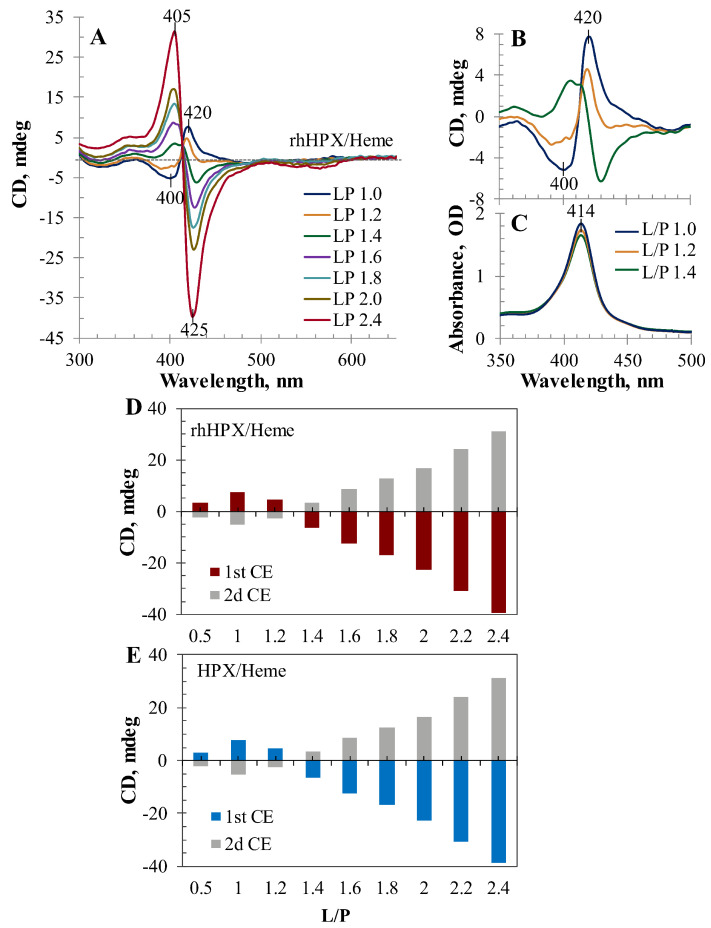
CD titration data for rhHPX and HPX with heme at higher than equimolar L/P. (**A**) Overlay of the induced CD (ICD) spectra of the rhHPX/Heme samples (L/P 1.0–2.4; for clarity, selected spectra are shown); (**B**) ICD spectra of rhHPX/Heme samples at L/P 1.0, 1.2, and 1.4, and (**C**) complementary UV/Vis spectra; (**D**,**E**) ICD spectral changes plotted as the observed 1st CE and observed 2nd CE versus increasing content of heme (L/P), respectively, for rhHPX (**D**) and plasma HPX (**E**).

Further CD titrations of rhHPX and plasma HPX with heme at higher heme-to-protein stoichiometric values (Figure 5) demonstrate dramatic ICD spectral changes related to the protein-bound chromophore. Figure 5A shows an overlay of the visible ICD spectra typical for rhHPX and plasma HPX with an increasing heme content in the titration samples (from an L/P 1.0 to 2.4; for clarity, only selected spectra are shown). First, it is noteworthy that all ICD spectra in the Soret region intercept through a classic isosbestic point, which is consistent with a Soret λ_max_ in the absorption spectra (414 nm). Second, the changes reflected by the ICD spectra for the samples with higher than equimolar heme content and the entire titration pattern (Figure 5A) differ from that observed at the lower heme content (Figure 4A) and demonstrate an inversion of the CD titration pattern per the increasing content of protein-bound heme. The most critical changes in the ICD spectra are observed for heme–protein samples as the ratio of L/P exceeds 1:1, which is visualized as complementary UV/Vis and CD spectra for the samples at an L/P of 1.0, 1.2, and 1.4 (Figure 5B,C). As shown in Figure 5B, an increase in heme content resulted in significant Soret CD changes with complete inversion of the bisignate ICD spectrum from the 1st positive (420 nm)/2nd negative (400 nm) blue trace at an L/P of 1.0 to the 1st negative/2d positive (green) trace at an L/P of 1.4 through an intermediate state (orange trace, L/P of 1.2). In the meantime, the complementary UV/Vis spectra reflected only relatively small increase of the Soret band absorbance intensity (Figure 5C).

A transition through an intermediate state with a shortened ICD amplitude (orange trace) and an inverse +/− ICD couplet is indicative of a strong opposite exciton coupling phenomenon with the 1st negative/2nd positive CEs. The ICD spectra at an L/P of 1.2 and 1.4 with changes in the ICD amplitude and signs are likely observed due to a superimposition of two opposite bisignate spectra.

Between an L/P of 1.4 and 2.4, the observed 1st negative/2nd positive ICD couplet strongly increased to a nearly conservative ICD spectrum. In comparison with the amplitude of the ICD associated with an L/P of 1.0, the magnitude of ICD amplitudes observed for the samples of a higher L/P (2.0 and 2.4) is indicative of a strong exciton coupling, suggesting an interplay between at least two heme chromophores.

To illustrate the inversion of the ICD pattern due to an arising opposite ICD couplet per the increased heme content in the samples, Figure 5D,E shows CD titration data plotted as the observed 1st CE and observed 2nd CE versus L/P for rhHPX and plasma HPX, respectively.

To the best of our knowledge, CD titrations have not been performed in any studies that would allow for an assessment of ICD changes dependent on the amount of protein-bound heme. By evaluating a wide range of heme-to-protein molar ratios (from as low as 0.05 to more than a two-fold molar excess), we approximate HPX interactions that may occur over a wide range of heme plasma concentrations consistent with those observed in health and disease. We believe that the Soret CD data collected for HPX–heme interactions at increasing heme concentrations, the differences defined between the data related to low and high heme concentration ranges, and the intriguing inversion of the ICD couplet suggest a novel complexity of heme’s interactions with HPX. The induced CD changes observed during HPX titration with heme are very likely the result of a few contributions from more than one heme chromophore in different protein chiral environments. From an analytical perspective, the bisignate positive/negative ICD pattern at the low L/P range, the inversion of the bisignate ICD at an L/P ratio greater than equimolar, along with the magnitude of the CD amplitude introduce novel findings. Since the observed changes are unambiguously dependent on the amount of HPX-bound heme, they can be best explained by exciton coupling between two identical heme chromophores on one HPX molecule.

Whether the observed strong CD couplet can be caused by the exciton interaction between hexa-coordinated low spin heme at the HPX primary binding site (which must be fully saturated at this level of the ligand) with the unbound heme or loosely associated at the protein surface is a very unlikely possibility, because to enable a strong through-space dipole–dipole interaction the chromophores must be properly oriented with respect to each other [45,46].

### 2.5. Heme Accessibility to Hydrogen Peroxide

**Figure 6:**
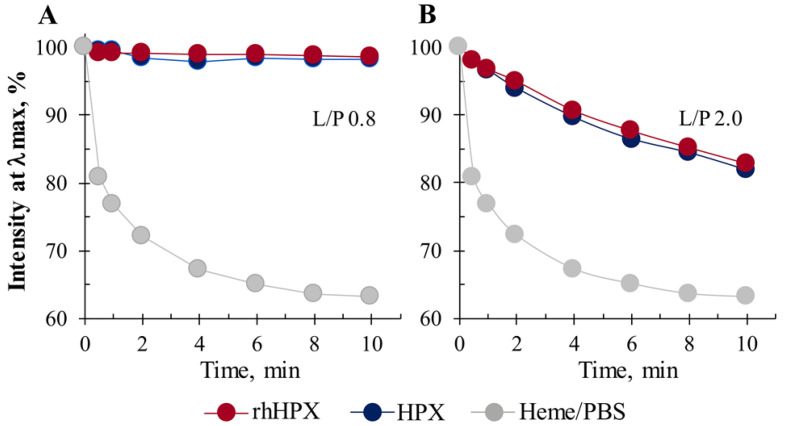
UV-Vis time-course measurements of heme complexes with plasma HPX and rhHPX in the presence of H_2_O_2_ (8-fold molar excess over the protein/heme complex) at an L/P of 0.8 (**A**) and 2.0 (**B**) versus heme in PBS alone.

We examined the rhHPX/Heme and HPX/Heme complexes for heme accessibility to hydrogen peroxide (H_2_O_2_) by using catalase-like activity assay, as described earlier [56]. As shown in Figure 6A for rhHPX/Heme and HPX/Heme complexes at an L/P of 0.8, the Soret band intensity at 414 nm was not affected up to 8-fold molar excess of H_2_O_2_, suggesting that the complexes remained stable and protein-bound heme was not being degraded. This observation is consistent with the biochemical interaction where HPX binds and locks heme in a bis-histidyl tweezer-fixed conformation, and the hexa-coordinated low spin heme is oxidatively inert and cannot interact with prooxidants, such as H_2_O_2_ [10,57]. However, heme accessibility to H_2_O_2_ in the complexes at higher L/P ratios demonstrated differing behavior. Figure 6B shows time-course measurements of heme complexes at an L/P of 2.0 in the presence of the same 8-fold access of H_2_O_2_. Prior to adding H_2_O_2_, the samples were subjected to Amicon filtration to remove any possible non protein-bound heme. 

The decline in the Soret band intensity is observed for both samples (B), but it is considerably different from that of heme in PBS alone (gray trace). These data are indicative of some heme bound to a protein low affinity secondary binding site in which heme is less protected, more accessible to the external environment, and therefore able to interact with H_2_O_2_.

### 2.6. Temperature-Induced CD Assessment

**Figure 7:**
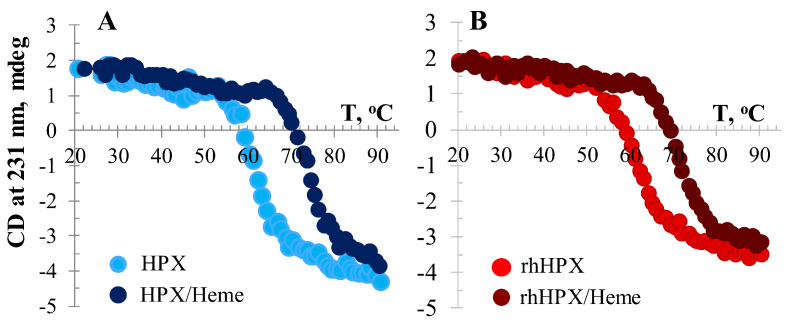
Temperature-induced CD changes: (**A**) Plasma HPX and HPX/Heme at an L/P of 1.0 and (**B**) rhHPX and rhHPX/Heme at an L/P of 1.0.

A temperature-induced CD study was performed to examine whether the conformational stability of rhHPX/Heme may differ from that of the recombinant protein apo-form (Figure 7). The ellipticity of samples was monitored at two wavelengths, 206 nm and 231 nm, within a temperature range spanning from 20 to 90 °C. Figure 7 shows the thermal unfolding (melting) data collected for protein apo-forms and heme-complexes at the wavelength 231 nm, which is usually regarded as a sensitive and reliable reporter of the HPX tertiary structure [37,38,39,40,41].

Temperature-induced CD data suggest that heme binding to rhHPX stabilizes the protein structure as indicated by a T_m_ = 71 ± 0.5 °C observed for the rhHPX/Heme complex (L/P of 1.0) in comparison with that of apo rhHPX (T_m_ = 62 ± 0.5 °C). Although the CD melting profiles for the recombinant and plasma samples are not identical, the melting points of the complexes approximate each other. In comparison with the data collected for apo-proteins, these results demonstrate that both rhHPX and plasma HPX are largely stabilized by bound heme. The T_m_ values determined here for plasma apo HPX and HPX/Heme appear to be slightly different than values obtained using differential scanning calorimetry [38]; nonetheless, our data are comparable to the T_m_ values determined by Rosell et al. from their temperature-induced CD study [58].

### 2.7. SEC-HPLC Assessment

SEC-HPLC was employed in this study to examine the molecular integrity of the recombinant and plasma HPX apo-forms and HPX-heme complexes at various L/P ratios.

**Figure 8:**
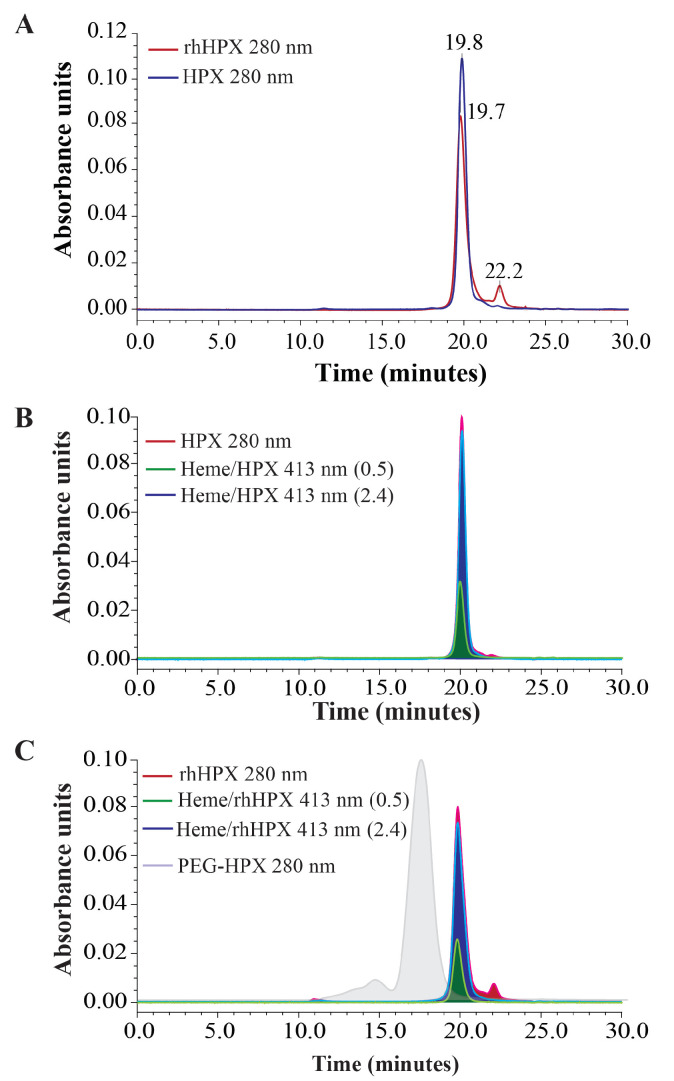
SEC-HPLC assessment: (**A**) Apo-proteins rhHPX and HPX; (**B**) HPX/Heme samples at an L/P of 0.5 and 2.0 vs. HPX; (**C**) Heme complexes with rhHPX (L/P of 0.5 and 2.0) vs. rhHPX and PEG-HPX. Detection of the peaks was performed at 280 nm and 413 nm as specified on the plots.

The SEC data shown in Figure 8A demonstrate overlapping rhHPX and plasma HPX peaks in the representative chromatograms, with retention times 19.8 min and 19.7 min, respectively. However, the elution of rhHPX was accompanied by a small peak with a retention time 22.2 min that was considered a protein impurity visualized at a protein detection wavelength of 280 nm. The intensity of this small peak did not increase or change per rhHPX binding with heme, suggesting a small non-heme binding impurity. According to the chromatograms overlaid in Figure 8B, the elution times of plasma HPX/Heme complexes of various heme content (shown for an L/P of 0.5 and 2.0) do not differ from that of plasma HPX apo-form. The same is shown for the respective rhHPX samples (plot C). In addition, Figure 8C provides an overlay with a chromatogram of a pegylated form of HPX (PEG-HPX), which we used as a reference high molecular weight sample to visualize a range (10 to 20 min) of possible heme-induced protein aggregation. Based on the SEC-HPLC data, no oligomers were identified for the rhHPX and HPX complexes with heme. This is consistent with earlier observations that heme binding to HPX did not prompt an aggregation [37,59]. Based on this observation, the possibility of heme-induced aggregation, which might be an alternative cause of bisignate CD patterns and/or inversion of the CD pattern (*vide supra*) is unlikely. Moreover, under SEC-HPLC conditions, any heme excess loosely associated with either rhHPX or plasma HPX is expected to be removed from the complexes; therefore, from a peak height or area quantitation corresponding to rhHPX/Heme at an L/P of 0.5 and 2.0 samples estimates of approximately a 1.5-fold molar equivalent of heme remain bound within the rhHPX/Heme sample. This contrasts with the expected equimolar L/P ratio and is suggestive of an additional, but likely low affinity heme binding site.

There remains the possibility that the characteristic bisignate ICD profile observed from the very first steps of HPX titration with heme may be caused by the dimeric state of the heme stock solution in DMSO (or NaOH) aliquots that were used for the HPX titration. Nonetheless, this scenario seems unlikely because similar heme stock solutions and aliquots were used in primary titration steps for human albumin and α1-microglobulin, both of which demonstrate a traditional one chromophore—one major Cotton Effect CD profile [56]. Future work should consider a thorough interpretation of the discrepancies between the crystal structure, establishing a single heme binding site, and the observed bisignate CD spectral data, which may be achieved using specifically designed HPX mutants.

The purpose of our study was to characterize rhHPX produced in the Expi293F^TM^ mammalian cell system for the interaction with heme and use this data as a reference for later experiments that evaluate novel HPX constructs (e.g., fusion proteins). Unlike previous CD studies that mainly characterized equimolar HPX-heme mixtures, we performed a titration of rhHPX (as well as plasma HPX) with heme across a wide range of the ligand concentrations, from the ligand-to-protein molar ratio 0.1 up to 2.4. The purpose was to evaluate the pattern of protein-induced CD of heme in rhHPX/Heme complexes that can be used as a reference for the characterization of heme binding to novel fusion HPX complexes that may exhibit a greater capacity for the ligand than HPX alone. Our thermal unfolding study demonstrated that heme binding stabilizes the protein structure (T_m_ 71 ± 0.5 °C vs. 62 ± 0.5 °C). The data generated by the titration of rhHPX and plasma HPX with heme, together with catalase-like activity and SEC-HPLC data, first demonstrate that rhHPX and plasma-derived HPX behave similarly, and second, that HPX interactions with heme are not limited to a single heme-binding site within the HPX plasma or recombinant forms.

According to the crystal structure, heme binds to HPX in a bis-histidine hexa-coordinated ligation mode being captured between His-213 and His-226 residues. The possibility of other heme-binding sites has been considered, partly based on molecular modeling, but it has been limited to an alternative heme-binding site of the highest heme-binding affinity [24,60]. Thus, a pair of His-82 and His-127 at the broader end of the central tunnel has been discussed [24,60]. Heme binding to this plausible site was logically proposed to occur in a bis-histidyl ligation mode. However, the data from amino-acid sequence homology comparisons of HPXs from different vertebrate species, as well as site-directed mutagenesis studies suggest that there is only one invariant His (i.e., His-127) residue capable of acting as a heme-accepting site [60]. Cox et al. also demonstrated that the environments of at least four of five His residues are perturbed by heme binding, and that the heme environments of intact HPX and domain I are not identical. Therefore, to further discuss the data generated in our study suggesting a secondary heme-binding site, we consider His-127 is a plausible residue for a non-bis-histidyl interaction as a secondary binding site of lower affinity. We speculate that a weaker ligation of heme to His-127 in coordination with other amino acid residues could reasonably account for our observations. Our data support a secondary site of low affinity, which can bind an additional amount of heme, thus enabling a strong exciton coupling between the heme chromophores. The methodology developed in this study, and specifically rhHPX/heme titration data with a characteristic induced CD pattern, which strongly depends on the heme concentration in the sample, are essential for interpretation of already reported data and for the characterization of novel HPX constructs. Further research will help to refine our conclusions and foster interpretations of the intriguing interactions between heme and HPX variants.

## 3. Material and Methods

### 3.1. Materials

Hemin, dimethylsulfoxide (DMSO), and hydrogen peroxide (H2O2) were purchased from Sigma Chemical Co. (St. Louis, MO, USA). Phosphate buffer saline (PBS) 10X (0.1 M phosphate buffer with 27 mM KCl and 1.37 M NaCl, pH 7.4) and deionized water were from GE Healthcare (Piscataway, NJ, USA). Buffers for rhHPX affinity purification were based on phosphate (20 mM) containing NaCl (500 mM) and increasing amounts of imidazole for equilibration (10 mM), washing (25 mM), and elution (500 mM). Size exclusion chromatography (SEC) was performed using PBS as mobile phase. Other chemicals used in this study were analytical grade from Fisher Scientific (Fair Lawn, NJ, USA).

### 3.2. Generation of rhHPX Expression Plasmid and Transient Transfections 

A cDNA encoding human hemopexin (Genbank accession number NP_000604) was codon-optimized for human expression and synthesized by Geneart^®^ (Invitrogen™, Thermo Fisher Scientific, Waltham, MA, USA) with a Kozak consensus sequence [61] (GCCACC) immediately upstream of the initiating methionine (+1) and ligated into pcDNA3.1 (Invitrogen™, Thermo Fisher Scientific). Large-scale preparations of plasmid DNA were carried out using QIAGEN Plasmid Giga Kits (12191) according to the manufacturer’s instructions. The nt sequences of the plasmid construct was verified by sequencing both strands using BigDye™ Terminator Version 3.1 Ready Reaction Cycle Sequencing (Invitrogen™, Thermo Fisher Scientific.4337455) and an Applied Biosystems 3130xl Genetic Analyzer.

Transient transfections of the rhHPX expression plasmid using Expi293F™ cells were performed using Expifectamine^™^ transfection reagent (Invitrogen, Life Technologies, Carlsbad, CA, USA) according to the manufacturer’s instructions. Cells were transfected at a final concentration of 1 × 10^6^ viable cells/mL and incubated in a shaking incubator for 6 days at 37 °C in 8% CO_2_. Pluronic F68 (GIBCO, Life Technologies), to a final concentration of 0.1% *v/v*, was added 4 h post-transfection. At 24 h post-transfection, cell cultures were supplemented with LucraTone Lupin (Millipore, Billerica, MA, USA) to a final concentration of 0.5% *v/v*. The cell culture supernatants were harvested by centrifugation at 2500 rpm and then were passed through a 0.45 μm filter (Nalgene, Rochester, NY, USA) prior to purification. 

### 3.3. Purification of rhHPX

rhHPX expressed using the Expi293F™ Expression System was subjected to an automated 2-step purification protocol on an ÄKTA Express system operated by Unicorn software (GE Healthcare, Milwaukee, WI, USA). The purification protocol was based on the described strong interaction of HPX with bivalent metal affinity chelate resins even in the absence of a His-Tag [34]. Briefly, 2 L of filtered conditioned media containing rhHPX was pre-concentrated to 220 mL using a tangential flow filtration system (ÄKTA Crossflow, GE Healthcare, Milwaukee, WI, USA) equipped with a 30 kDa cut-off membrane. The retentate was buffer-exchanged into PBS to overcome the potential interference of the Expi293 media components with the IMAC matrix. Then, conditioned media was loaded at 0.3 mL/min onto an equilibrated His-Trap column (5 mL, GE-Healthcare) followed by sequential washing with equilibration buffer and wash buffer at 0.5 mL/min. Bound rhHPX was eluted using elution buffer and further purified by SEC using a Superdex200 26 × 60 column (ÄKTA Crossflow, GE Healthcare, Milwaukee, WI, USA) equilibrated with PBS. The main peak was collected and concentrated to 10.5 mg/mL using an Amicon Ultra-15 Centrifugal Ultracel 10K filter (Millipore, Darmstadt, Germany) according to the manufacturer’s instructions. The concentration of the purified rhHPX was measured using the Trinean DropPlate system as previously described [62], and the purity of rhHPX was confirmed by analytical SEC and SDS-PAGE.

### 3.4. PEG-HPX Preparation

PEG-HPX was prepared from plasma-derived HPX provided by CSL Behring, Kankakee, IL, USA. HPX was further modified using Maleimide NHS-activated PEG (Thermo Fisher Scientific, Basel, Switzerland) according to the manufacturer’s instructions. The approximate weight range (120–180 kDa) was determined by size exclusion chromatography.

### 3.5. Stock Solutions of Heme, rhHPX, and Plasma HPX

Heme stock solutions in DMSO (1.4 mM and 0.7 mM) were freshly prepared prior to use, purged with argon, and kept protected from light. The heme concentrations in the stock solutions were determined spectrophotometrically using heme molar extinction coefficient of 170,000 M^−1^ cm^−1^ at λ_max_ 404 nm [63]. Stock solutions of apo-proteins (plasma HPX and rhHPX) were prepared in PBS, pH 7.4, and diluted to a concentration of 19 μM (for heme-binding experiments) or 4 μM (for far-UV CD assessment). Protein concentrations were measured spectrophotometrically using extinction coefficient of 120,000 M^−1^ cm^−1^ at 280 nm published for human apo-HPX [64].

### 3.6. Titration of Plasma HPX and rhHPX with Heme

Titration of each apo-protein with heme was performed using 19 μM rhHPX (or plasma HPX) samples in PBS. Calculated aliquots of heme stock solution in DMSO (0.7 mM—for low L/P range, and 1.4 mM—for higher L/P range) were added stepwise to 1.5 mL of each protein solution in a 10 mm pathlength quartz cuvette. Each solution was gently mixed (by turning the cuvette upside-down), followed by UV/Vis and CD monitoring until no significant spectral changes indicate a completion of the protein/heme interactions. The complementary CD and UV/Vis spectra were collected (for each L/P step), followed by addition of the next heme increment, and so on. During titration, the samples were maintained at 20 ± 1 °C, protected from light, and flashed by argon. Initial apo-protein stock solutions served as a blank in spectral measurements. A total amount of DMSO in the titration samples did not exceed 4% *v/v*.

### 3.7. UV/Vis Measurements

UV/Vis electronic absorption measurements were performed on an Agilent HP 8453 UV-visible spectrophotometer (Agilent Technologies Deutschland GmbH, Waldbronn, Germany) at 25 ± 0.2 °C in a wavelength range 250–700 nm using a quartz cuvette of 1 cm pathlength. The HPX/heme titration samples with absorbance intensity close to or beyond the instruments upper limit (2.4 OD) were measured in a 5 mm pathlength quartz cuvette, and the results were doubled. To collect for UV/Vis differential spectra of the HPX/heme complexes, the apo-protein sample (rhHPX or plasma HPX) was first measured as a blank. 

### 3.8. CD Measurements

CD measurements were performed on a Jasco J-815 Spectropolarimeter (JASCO Co., Tokyo, Japan) at 25 ± 0.2 °C and the temperature was maintained by a Peltier thermostat. The spectra were measured in triplicate with a scan speed of 100 nm/min, bandwidth of 1.0 nm, and resolution of 0.2 nm. The far-UV CD spectra were recorded between 180 and 260 nm in a 1 mm quartz cuvette. Protein concentration in the samples for far-UV CD was 4.5 μM in PBS, pH 7.4. The baseline was subtracted by using PBS as a blank. The unsmoothed CD spectra of protein samples were converted into Δε and analyzed for the secondary structure elements using CDPro/CONTIN.

The induced CD spectra of rhHPX and plasma HPX titration with heme were recorded between 300 and 650 nm in a rectangular quartz cuvette with 1 cm pathlength. Induced CD was determined as the CD of the protein–heme sample measured versus protein alone as a blank. The volumes, protein final concentration (18 μM), and the content of DMSO (4%) for all samples were kept constant. An ellipticity of CD spectra was expressed in millidegrees (mdeg).

Temperature-induced conformational changes were assessed for rhHPX, HPX, and the respective heme-protein L/P 0.9 complexes at the protein concentration approximately 4.5 μM in PBS, pH 7.4. The ellipticity at 231 nm was recorded as a function of temperature from 20 to 90 °C with a temperature increase 2 °C/min as controlled by the Peltier device connected to a Jasco J-815 spectropolarimeter. 

### 3.9. Stability to Hydrogen Peroxide

An aliquot of H_2_O_2_ solution was added to a 1 mL sample of 19 μM rhHPX/Heme or HPX/Heme (L/P 0.8) to create 8-fold molar excess over the complex, and the mixture was immediately monitored by UV/Vis for 10 min at room temperature. The rhHPX/Heme or HPX/Heme complexes at an L/P of 2.0 were first subjected to two rounds of Amicon 3K filtration, followed by Soret band absorption measurements and then treated with H_2_O_2_ using the same procedure. A reference heme sample in PBS was freshly prepared (due to limited stability in aqueous solutions) and adjusted to ~0.6 OD at λ_max_ 363 nm. After mixing with an aliquot of H_2_O_2_ (8-fold excess), the sample was immediately UV/Vis monitored. The time-course UV/Vis measurements were taken at room temperature in a 10 mm pathlength quartz cuvette at the following time points: 30 s, 1, 2, 4, 6, 8, and 10 min. The concentration of H_2_O_2_ was determined spectrophotometrically as described elsewhere [65].

### 3.10. SEC-HPLC

Molecular integrity was evaluated by using a BioSep-SEC-s3000 (600  ×  7.5 mm) column (Phenomenex, Torrance, CA, USA) connected to a Waters 2535 Quaternary Gradient Module pump and Waters 2998 Photodioide Array Detector controlled by a Waters 600 controller using Empower 2 software (Waters, Milford, MA, USA). Mobile phase: 1 mM potassium phosphate (K_2_HPO_4_/KH_2_PO_4_) pH 7.4. The injection volume of each protein solution was 50 μL. Detection was performed at three wavelengths: 280, 404, and 414 nm.

## 4. Conclusions

In this study, we characterized rhHPX for heme binding using different analytical techniques in comparison with plasma-derived HPX. Heme–protein interactions were evaluated across a wide range of heme concentrations from a ligand-to-protein molar ratio 0.1 up to 2.4 to make the outcomes of this study useful for the evaluation of heme binding to HPX-fusion proteins produced in the same system. Our UV/Vis, CD, SEC-HPLC, and catalase-like activity assay data demonstrated a close similarity between rhHPX and the plasma counterpart. Thermal unfolding studies demonstrated that heme binding greatly stabilizes the protein structure. The induced CD data revealed dramatic Soret CD changes that are dependent on the amount of protein-bound heme. These data suggested that HPX interactions with heme are not limited to a single heme-binding site. The data generated in this study provide a novel interpretation of heme interactions with HPX and offer a unique understanding of HPX-ligand binding reported on for the first time. We further suggest that the techniques reported herein provide a useful strategy to evaluate heme binding in novel HPX constructs.

## Data Availability

All data will be made available upon request.

## Abbreviations

CD	Circular dichroism
CE	Cotton effect
DMSO	Dimethylsulfoxide
EC	Exciton coupling
HA	Human albumin
HA/Heme	Methemalbumin, HA/Heme complex
Heme	Iron-protoporphyrin IX
HPX	Hemopexin
HPX/Heme	Hemopexin/Heme complex
L/P	Ligand-to-protein (molar) ratio
λ_max_	Wavelength of absorption maximum
PBS	Phosphate buffer saline
SEC-HPLC	Size-exclusion high-performance liquid chromatography
